# Functional characterization of neutrophils in patients with multiple myeloma

**DOI:** 10.1371/journal.pone.0360063

**Published:** 2026-10-08

**Authors:** Kapina Mwampembwa, Emmanuel Mweni, Humphrey Mwape, Ruth Daka, John Nzobokela, Chrispin Chisanga, Sody Munsaka

**Affiliations:** 1 Department of Biomedical Sciences, School of Health Sciences, University of Zambia, Lusaka, Zambia; 2 Haematology Section, Pathology Department, the University Teaching Hospital, Lusaka, Zambia; 3 Pathology Department, Ndola Teaching Hospital, Ndola, Zambia; Stellenbosch University Faculty of Medicine and Health Sciences, SOUTH AFRICA

## Abstract

Multiple myeloma is a hematologic cancer caused by abnormal plasma cell proliferation in the bone marrow and is associated with significant immune dysfunction. Neutrophils, essential components of the innate immune response, may function poorly in patients with multiple myeloma, increasing the risk of infection and affecting disease progression. This study aimed to systematically evaluate the extent of neutrophil dysfunction in patients with newly diagnosed and relapsed multiple myeloma. The study employed a cross-sectional analytical design and recruited 72 participants: 30 multiple myeloma patients and 42 age- and sex-matched healthy controls from the University Teaching Hospital and the Cancer Diseases Hospital of Lusaka, Zambia. 4 mL of venous blood was collected from all participants in ethylenediaminetetraacetic acid vacutainers for laboratory analysis. Neutrophils were isolated from whole blood using Polymorphprep™, and functionality was assessed using in vitro assays, including a phagocytosis assay using fluorescently labeled bacterial particles and a reactive oxygen species assay using the fluorescent probe 2’, 7’-Dichlorodihydrofluorescein diacetate, measured by flow cytometry. Statistical analysis was performed using STATA. Neutrophils from multiple myeloma patients demonstrated significantly lower phagocytic capacity (45.0% vs 75.9%, P < 0.001) and reactive oxygen species production (44.7% vs 74.9%, P < 0.001) compared to controls. The logistic regression analysis demonstrated strong associations between multiple myeloma and both reactive oxygen species (OR=5097.6, P < 0.001) and phagocytic dysfunction (OR=7272.7, P < 0.001). By contrast, age, sex, white blood cell count, and absolute neutrophil count were not significantly associated with these outcomes. The study found that patients with multiple myeloma exhibit qualitative impairment of neutrophil function, despite normal quantitative neutrophil counts, underscoring the importance of functional immune monitoring in patient management.

## Introduction

Multiple myeloma (MM) is a clonal plasma cell malignancy with an increasing incidence worldwide [1,2]. This trend is evidenced by an increase in newly diagnosed cases, rising from an estimated 159,985 in 2018 [3] to 176,404 in 2020 [4]. It accounts for 1% of all cancers and approximately 10% of all hematologic malignancies [5–7]. The disease is characterized by uncontrolled proliferation of plasma cells in the bone marrow and the production of monoclonal immunoglobulins [8,9]. Beyond its malignant course, MM profoundly disrupts immune homeostasis, leaving patients highly vulnerable to infections, which remain a leading cause of morbidity and mortality [10–12].

Neutrophils are central to innate immunity, providing rapid antimicrobial defense through phagocytosis, reactive oxygen species production, and receptor-mediated signaling [13–19]. In MM, however, disease-driven alterations in the bone marrow microenvironment, compounded by treatment effects, may impair neutrophil function. Several studies have documented that neutrophils in MM exhibit altered surface marker expression [9,20,21], impaired neutrophil extracellular traps (NETs) [22,23], and dysregulated cytokine production, including increased levels of interleukin-6 (IL-6), vascular endothelial growth factor (VEGF), and tumor necrosis factor-alpha (TNF-α) among others [24–26], These cytokines regulate immune cell behavior, and because neutrophils are highly responsive to cytokines, chronic exposure to this inflammatory environment might alter their phenotype and function. However, few have examined phagocytic capacity and reactive oxygen species production as measures of neutrophil dysfunction. These functional parameters are critical, as impaired neutrophil activity may contribute to the raised predisposition to infections observed in this patient population, underscoring the need for in-depth assessment of neutrophil dysfunction in the context of disease pathophysiology.

Studying neutrophil function in patients with MM is important for understanding immune dysregulation and infection risk, given the association between MM and impaired immunity [16,27]. Neutrophils play a central role in defending against infections. Characterizing phagocytic capacity and reactive oxygen species production helps define the extent of neutrophil dysfunction and may explain the increased susceptibility to infections observed in affected patients.

Evaluating key neutrophil effector functions may provide insight into the contribution of neutrophil impairment to infection susceptibility in patients with MM and may help inform supportive care strategies. This study, therefore, investigated neutrophil function using established phagocytic and reactive oxygen species assays to define immune dysregulation and its implications for infection risk.

## Materials and methods

### Study design

This cross-sectional analytical study was conducted at the University Teaching Hospital (UTH), comprising Adult Hospital and Cancer Diseases Hospital (CDH), Lusaka, Zambia, between 17 November 2025 and 3 April 2026. UTH is Zambia’s largest referral and teaching hospital and the country’s main center for specialized care and medical education. CDH is Zambia’s national referral center for oncology care and treatment. Located within the UTH complex, it serves as the country’s primary facility for cancer diagnosis, radiotherapy, chemotherapy, and palliative care.

Peripheral blood samples (4 mL) from participants were collected once from MM patients and age- and sex-matched healthy controls. Neutrophil function was assessed by measuring absolute neutrophil count (ANC), phagocytic activity, and reactive oxygen species production using flow cytometry.

### Study population

The study participants included MM patients and age- and sex-matched healthy individuals. Eligible MM patients were ≥18 years with a confirmed diagnosis (newly diagnosed, relapsed, or refractory). Healthy controls had no history of malignancy or immune-related disorders. Exclusion criteria included acute infections, chronic inflammatory or autoimmune conditions, uncontrolled metabolic disorders, and use of corticosteroids or cytotoxic immunosuppressants.

### Sample size determination

The sample size was calculated to compare neutrophil functional parameters between patients with MM and healthy controls. The primary outcomes were ANC, phagocytic activity, and reactive oxygen species production, treated as continuous variables in two independent groups. Using formula for comparing two independent means [28–30], and assuming a 95% confidence level with 80% power, the minimum required sample size was 72 participants per group. Due to recruitment limitations, the final sample comprised 30 patients with MM and 42 healthy controls. The effective sample size was then recalculated using the harmonic mean formula [31], yielding 35 participants per group. Based on this achieved sample size, the minimum detectable effect size was Cohen’s d = 0.67, indicating adequate power to detect moderate-to-large differences in neutrophil functionality between groups.

### Sampling technique

#### Multiple myeloma patients recruitment.

MM patients were recruited using consecutive sampling, enrolling all eligible patients presenting at CDH during the study period. All individuals aged ≥18 years with a confirmed diagnosis of MM were considered eligible. Screening involved medical history reviews, clinical records, and diagnostic confirmation forms to verify eligibility. Newly diagnosed MM was defined according to the International Myeloma Working Group (IMWG) diagnostic criteria, which require at least 10% clonal bone marrow plasma cells or a biopsy-proven bony or extramedullary plasmacytoma, in addition to at least one myeloma-defining event such as hypercalcemia, renal impairment, anemia, or bone lesions [32]. Disease stage was determined using the International Staging System (ISS), based on serum β2-microglobulin concentrations, with classification into ISS Stage I, II, or III according to established criteria [33]. Exclusion criteria included acute infections, chronic inflammatory or autoimmune conditions, uncontrolled metabolic disorders, and current use of prolonged corticosteroid or cytotoxic immunosuppressant therapy. Of the 32 patients initially identified, 2 were excluded (1 with a chronic inflammatory condition and 1 with an uncontrolled metabolic disorder). The final MM group comprised 30 participants, all of whom were not receiving anti-myeloma treatment at the time of enrollment.

#### Healthy control recruitment.

For selecting healthy controls, a simple random sampling technique was used to ensure no bias in the sampling frame. Potential controls were identified through hospital staff, blood donor registers, and health screening programs. A master eligibility list was developed, and a computer-generated sequence of random numbers was used to select individuals for the study. After ascertaining their eligibility and obtaining their consent, data on demographic and clinical variables were recorded, and blood samples were taken. In total, 53 healthy individuals were screened. 11 were excluded (8 on corticosteroids and 3 with uncontrolled metabolic disorders). The final control group comprised 42 participants, matched to the patient cohort by age and sex distribution. Participant recruitment and exclusions are summarized in Fig 1.

**Fig 1:**
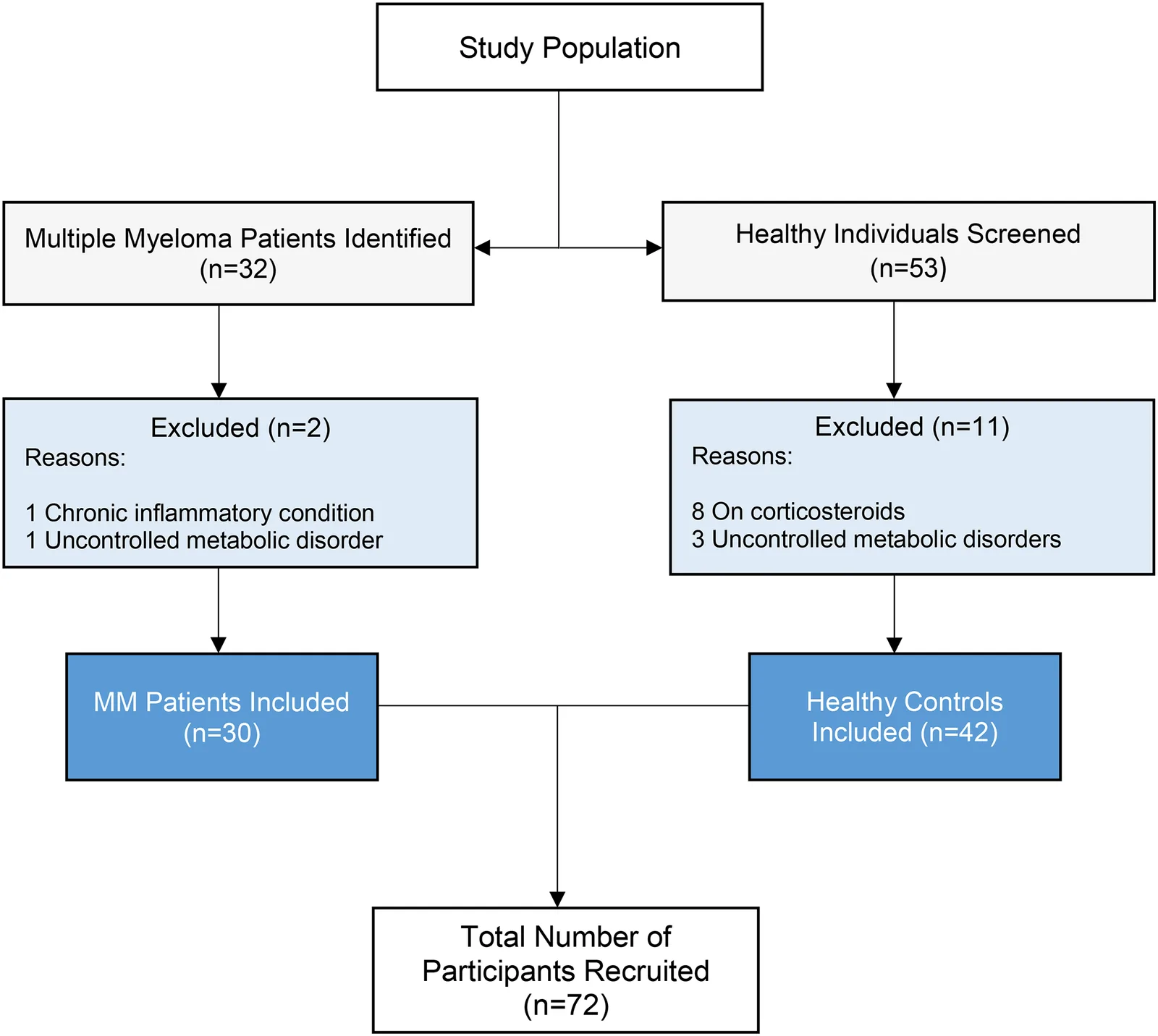
Recruitment and Sampling Summary of Multiple Myeloma Patients and Healthy Controls.

### Experimental approach

#### Sample collection and handling.

Venous blood samples (4 mL) were collected from participants using sterile ethylenediaminetetraacetic acid (EDTA) vacutainers. Immediately after collection, each tube was gently inverted 8–10 times to mix the blood with the anticoagulant, maintain sample stability, and prevent clot formation. To preserve specimen quality and support accurate hematological analysis, all samples were processed within 2–4 hours of collection. During this period, samples were stored upright at room temperature and handled carefully to avoid hemolysis. By following these procedures, pre-analytical errors were minimized, and the reliability of subsequent measurements was enhanced.

#### Determination of absolute neutrophil count.

Upon arrival in the hematology/immunology laboratory, EDTA blood samples were received, verified for correct labeling, and gently inverted before analysis. The Sysmex XN-1000 hematology analyzer was verified for calibration status, and quality control checks were performed using manufacturer-supplied control materials to ensure accuracy. Blood tubes were then loaded into the analyzer’s sample carousel, where the system automatically aspirated the required volume for testing. The instrument measured total white blood cell counts (WBC) expressed as ×10^9^/L, provided differential leukocyte percentages, and calculated absolute counts of neutrophils, lymphocytes, monocytes, eosinophils, and basophils. Using advanced fluorescence and flow cytometry principles, the analyzer differentiated cell populations with high precision, minimizing operator bias. Results were generated in real time, displayed on the instrument screen, and printed on A4 reports.

#### Isolation of neutrophils from whole blood.

Following determination of ANC, neutrophils were isolated from whole blood using Polymorphprep™, a sterile, ready-made density gradient medium specifically designed for isolating human polymorphonuclear leukocytes, primarily neutrophils. Polymorphprep™ and phosphate-buffered saline (PBS) were removed from the refrigerator and allowed to warm to room temperature before use. Whole blood was diluted 1:1 with PBS, and 4 mL of the diluted blood was slowly layered over 4 mL of Polymorphprep™ while the tube was tilted to prevent mixing. The samples were centrifuged at 3000 rpm for 30 minutes at room temperature with the brake off. The neutrophil layer was collected from between the mononuclear cell layer and the red blood cell pellet after centrifugation, washed twice with PBS, resuspended in 5 mL PBS, and assessed for viability by trypan blue exclusion before downstream functional evaluation.

A small aliquot of the cell suspension was mixed 1:1 with trypan blue and counted using a hemocytometer. Viable neutrophils excluded the dye and remained unstained, whereas non-viable cells appeared blue. Viability percentage was calculated as (number of viable cells)/(total cells)×100, and more than 90% viability was confirmed before downstream functional evaluation.

The purity of isolated neutrophils was assessed by microscopic examination of Wright-Giemsa-stained direct smear preparations. A total of 100 cells were counted under oil immersion, and the proportion of neutrophils was calculated. Neutrophil purity was defined as the percentage of neutrophils among the 100 counted cells, and exceeding 90% was considered acceptable for downstream functional assays.

### Phagocytosis assay

Isolated neutrophils were incubated with pHrodo™ Red *E. coli* BioParticles™ at 37 °C for 15 minutes to allow phagocytic uptake. Two tubes containing 100 μL of neutrophils resuspended in PBS and 20 μL of pHrodo™ BioParticles™ conjugate were prepared; one was placed on ice as the negative control, and the other was incubated in a water bath at 37 °C for 15 minutes as the positive control. After incubation, the tube from the water bath was removed and placed on ice to stop phagocytosis. Four controls were included: two negative and two positive. One negative control set was placed on ice, and the other positive control set was incubated at 37 °C in the water bath. 1 mL of the manufacturer’s buffer was added to each tube, briefly vortexed, and incubated for 5 minutes at room temperature. Samples were centrifuged at 3000 rpm for 5 minutes at room temperature. The supernatant was discarded, and each cell pellet was resuspended in 1 mL of the manufacturer’s wash buffer before acquisition on a CytoFlex flow cytometer. At least 15,000 neutrophil events per sample were collected. On the CytoFlex, phagocytic activity was determined by gating neutrophils on forward scatter/side scatter (FSC/SSC) (S1 Fig), applying a fluorescence threshold in the phycoerythrin (PE) channel (excitation 560 nm/emission 585 nm), and calculating the percentage of events classified as fluorescence-positive (S2 Fig). Debris was excluded by FSC thresholding, and doublets were removed using FSC-Area versus FSC-Height plots. Since a single fluorochrome was used to detect phagocytic activity, fluorescence compensation was not required.

#### Reactive oxygen species assay.

In a reactive oxygen species assay, isolated neutrophils were first activated by exposure to 1 × 10^7^/mL heat-killed *E. coli* (ATCC 25922) to induce reactive oxygen species production. Following activation, cells were collected and washed twice with PBS to remove residual bacteria. A working solution of 2’-7’-Dichlorodihydrofluorescein diacetate (DCFH-DA) was freshly prepared by diluting the 10 mM stock in serum-free medium to a final concentration of 10 µM. The neutrophils were incubated in 1 mL of 10 μM DCFH-DA working solution at 37 °C in the dark for 30 minutes, with gentle mixing every 5 minutes to ensure uniform probe uptake. After incubation, excess probe was removed by washing the cells twice with 1 mL of PBS, followed by resuspension in 1 mL of PBS. Reactive oxygen species levels were quantified by measuring 2’, 7’-dichlorofluorescein (DCF) fluorescence using a CytoFlex flow cytometer. Two controls were included: a positive and a negative. The positive control was prepared by diluting manufacturer-provided Rosup in serum-free medium at a 1:1000 ratio (1 mL serum-free medium plus 1 μL Rosup) to confirm the presence of a detectable reactive oxygen species signal. For the negative control, no additional reagent was added to the neutrophils before incubation, thereby establishing the baseline fluorescence signal. A minimum of 15,000 neutrophil events were acquired per sample. On the CytoFlex, reactive oxygen species production was determined by gating neutrophils on FSC/SSC, applying a fluorescence threshold in the fluorescein isothiocyanate (FITC) channel (excitation 488 nm/emission 525 nm), and calculating the percentage of events classified as fluorescence-positive. Debris was excluded by FSC thresholding, and doublets were removed using FSC-Area versus FSC-Height plots (S3 Fig). As a single fluorochrome was used for reactive oxygen species detection, fluorescence compensation was not required.

#### Definition of neutrophil dysfunction.

Neutrophil dysfunction was defined as phagocytic activity and/or reactive oxygen species production below the lower limit of normal, corresponding to values less than the 5^th^ percentile of healthy controls or more than 2 standard deviations below the mean of healthy controls.

#### Quality control.

Daily calibration was performed using CytoFlex calibration beads. Fluorescence minus one (FMO) controls were included to verify gating accuracy and minimize false-positive events. Single-stained controls were used to establish compensation matrices. A minimum of 15,000 neutrophil events were acquired per sample. Representative plots of gating strategy, phagocytosis, and reactive oxygen species assays are provided in Supplementary figures (S1–S3 Fig).

### Data analysis

The data analysis was structured in phases to address the investigation objectives and answer the key research questions on neutrophil function and its relationship to disease characteristics.

#### Data preparation and cleaning.

Initial data preparation involved importing the dataset, which contained information on 72 participants (30 MM patients and 42 healthy controls), from Microsoft Excel into STATA and GraphPad Prism. Data cleaning included checking for missing values, verifying data entry accuracy, and ensuring all variables were properly coded. Categorical variables such as sex, disease stage (I, II, III), disease status (newly diagnosed, relapsed, refractory), treatment status, and infection history were coded numerically for analysis. Continuous variables, including age, WBC, ANC, percentage of reactive oxygen species-positive neutrophils, and percentage of phagocytosis, were checked for outliers and normality using descriptive statistics and visual inspection.

#### Descriptive statistical analysis.

Descriptive statistics were calculated for all variables. For categorical variables, frequencies and percentages were presented in contingency tables. For continuous variables, measures of central tendency (mean, median) and dispersion (standard deviation, interquartile range, range) were calculated. Demographic characteristics were stratified by group (MM versus control) and, within the MM group, further subdivided by disease stage, disease status, treatment status, and infection history. This grouping allowed examination of how patient characteristics varied across subgroups.

#### Comparative analysis between groups.

The primary comparison focused on outcome variables between MM patients and healthy controls. For continuous, normally distributed variables, independent t-tests were used to compare means between the two groups. Statistical significance was set at p < 0.05, with highly significant results defined as p < 0.001. Chi-square tests or Fisher’s exact tests, when cell counts were small, were used to compare categorical variables between groups. Effect sizes were calculated using Cohen’s d to quantify the magnitude of differences, with interpretations following standard conventions (0.2 = small, 0.5 = medium, 0.8 = large, > 1.2 = very large).

Within the MM cohort, one-way analysis of variance (ANOVA) compared outcome variables across disease stages and disease status categories. For variables with only two categories, such as infection history, independent t-tests were used.

#### Logistic regression analysis.

Multivariate logistic regression models were constructed to assess predictors of neutrophil dysfunction. Independent variables included MM diagnosis, age, sex, WBC, and ANC. Model performance was measured using Nagelkerke R^2^, classification rate, and the Hosmer-Lemeshow goodness-of-fit test. Odds ratios with 95% confidence intervals were calculated to quantify associations between predictors and outcomes.

#### Post Hoc analysis.

Post hoc power analysis was conducted to assess statistical power for the primary outcomes, and Cohen’s d effect sizes with 95% confidence intervals were calculated to quantify the magnitude and precision of the observed group differences. Achieved statistical power was estimated using G-Power version 3.1 based on the observed effect sizes, actual sample sizes (30 MM patients and 42 healthy controls), two-tailed independent-samples t-tests, and a significance level of α = 0.05.

#### Statistical software and reporting.

All analyses were conducted systematically with results presented in comprehensive tables. Statistical significance levels were consistently reported, and confidence intervals were provided for effect estimates to facilitate interpretation of clinical significance alongside statistical significance. The analytical approach ensured a robust examination of patterns of neutrophil dysfunction while accounting for potential confounding variables and disease characteristics that might influence neutrophil function in patients with MM.

## Ethics

Ethical approval was obtained from the University of Zambia Biomedical Research Ethics Committee (UNZABREC reference number – 7234−2025), the Zambia National Health Research Authority (NHRA reference number – 2876−2025), and UTH management. Written informed consent was obtained from all participants. Confidentiality was maintained through coded identifiers, and blood collection was performed under aseptic conditions by trained personnel.

## Results

A total of 72 participants were enrolled in this study, including 30 patients with MM and 42 healthy controls. In the MM group, 20 patients were male (66.7%), and 10 were female (33.3%); in the control group, 21 were male (50.0%) and 21 were female (50.0%) (p = 0.172). The mean age was 51.8 ± 11.8 years in the MM group (range: 32–71 years, median: 52.5 years) and 48.6 ± 15.8 years in the control group (range: 23–72 years, median: 51.0 years) (p = 0.339).

Among MM patients, 7 (23.3%) had Stage I disease, 13 (43.3%) had Stage II disease, and 10 (33.3%) had Stage III disease. Regarding disease status, 19 patients (63.3%) were newly diagnosed, 8 (26.7%) had relapsed disease, and 3 (10.0%) had refractory disease. No patient (0.0%) was receiving active treatment at enrolment. Recent infection within the last month was documented in 24 MM patients (80.0%) and in none of the control participants (0.0%) (p < 0.001). Demographic characteristics of the study participants are summarized in Table 1.

**Table 1:** Demographic characteristics of study participants.

Characteristic	Multiple Myeloma	Control Group	Total
	(n = 30)	(n = 42)	(N = 72)
**Sex, n (%)**			
Male	20 (66.7)	21 (50.0)	41 (56.9)
Female	10 (33.3)	21 (50.0)	31 (43.1)
Age (years)			
**Mean ± SD**	51.8 ± 11.8	48.6 ± 15.8	49.9 ± 14.3
Median (IQR)	52.5 (42.3-60.8)	51.0 (33.0-62.8)	52.0 (36.0-62.0)
Range	32-71	23-72	23-72
**Age Groups, n (%)**			
20-39 years	5 (16.7)	11 (26.2)	16 (22.2)
40-59 years	17 (56.7)	19 (45.2)	36 (50.0)
≥60 years	8 (26.7)	12 (28.6)	20 (27.8)
**Disease Stage, n (%)**			
Stage I	7 (23.3)	N/A	7 (9.7)
Stage II	13 (43.3)	N/A	13 (18.1)
Stage III	10 (33.3)	N/A	10 (13.9)
**Disease Status, n (%)**			
Newly diagnosed	19 (63.3)	N/A	19 (26.4)
Relapsed	8 (26.7)	N/A	8 (11.1)
Refractory	3 (10.0)	N/A	3 (4.2)
**Treatment Status, n (%)**			
On Treatment	0 (0.0)	N/A	0 (0.0)
Not on Treatment	30 (100.0)	42 (100.0)	72 (100.0)
**Infection History, n (%)**			
Recent infection (last month)	24 (80.0)	0 (0.0)	24 (33.3)
No recent infection	6 (20.0)	42 (100.0)	48 (66.7)

Within the MM participants, mean age did not differ significantly across disease stages (Stage I: 47.1 ± 12.4 years, Stage II: 51.2 ± 10.9 years, Stage III: 55.6 ± 12.5 years; p = 0.264), disease status categories (newly diagnosed: 52.1 ± 11.9 years, relapsed: 51.8 ± 13.2 years, refractory: 48.3 ± 10.7 years; p = 0.891), infection history (recent infection: 52.3 ± 12.3 years, no recent infection: 49.7 ± 10.0 years; p = 0.600). Table 2 summarizes the age distribution of MM patients by disease characteristics.

**Table 2:** Age distribution by disease characteristics (multiple myelomas only, n = 30).

Characteristic	n	Mean Age ± SD	Median (Range)	p-value
**Disease Stage**				0.264^a^
Stage I	7	47.1 ± 12.4	45.0 (32-67)	
Stage II	13	51.2 ± 10.9	50.0 (32-67)	
Stage III	10	55.6 ± 12.5	58.5 (40-71)	
**Disease Status**				0.891^a^
Newly diagnosed	19	52.1 ± 11.9	54.0 (32-71)	
Relapsed	8	51.8 ± 13.2	50.5 (39-67)	
Refractory	3	48.3 ± 10.7	45.0 (40-60)	
**Infection History**				0.600^b^
Recent infection	24	52.3 ± 12.3	53.5 (32-71)	
No recent infection	6	49.7 ± 10.0	47.0 (34-67)	

a: Analysis performed using One-way ANOVA; b: An independent t-test was applied.

WBC was 4.16 ± 0.96 × 10^9^/L (median: 4.04, range: 2.87–6.67) in MM patients and 4.02 ± 0.87 × 10^9^/L (median: 3.94, range: 2.48–6.67) in controls, with a mean difference of +0.14 × 10^9^/L (95% CI: −0.31 to 0.59, p = 0.532, Cohen’s d = 0.15). ANC was 1.98 ± 0.21 × 10^9^/L (median: 1.96, range: 1.71–2.32) in MM patients and 2.04 ± 0.36 × 10^9^/L (median: 2.01, range: 1.28–2.82) in controls, with a mean difference of −0.06 × 10^9^/L (95% CI: −0.20 to 0.08, p = 0.403, Cohen’s d = 0.20). The percentage of reactive oxygen species positive neutrophils was 44.7 ± 8.4% (median: 42.5, range: 29.9–75.2) in MM patients and 74.9 ± 6.7% (median: 76.0, range: 58.2–85.1) in controls, with a mean difference of −30.2% (95% CI: −33.5 to −26.9, p < 0.001, Cohen’s d = 3.91). Phagocytic activity was 45.0 ± 10.2% (median: 42.1, range: 35.2–82.1) in MM patients and 75.9 ± 5.5% (median: 78.4, range: 63.4–84.9) in controls, with a mean difference of −30.9% (95% CI: −35.0 to −26.8, p < 0.001, Cohen’s d = 3.65). Table 3.

**Table 3:** Comparison of hematological parameters and neutrophil functional characteristics between multiple myeloma patients and healthy controls.

Outcome Variable	Multiple Myeloma (n = 30)	Control Group (n = 42)	Mean Difference (95% CI)	p-value	Cohen’s d
**WBC (x10^9^/L)**					
Mean ± SD	4.16 ± 0.96	4.02 ± 0.87	0.14	0.532^a^	**0.15**
Median (IQR)	4.04 (3.58-4.55)	3.94 (3.52-4.40)	(−0.31 to 0.59)		
Range	2.87-6.67	2.48-6.67			
**ANC (x10^9^/L)**					
Mean ± SD	1.98 ± 0.21	2.04 ± 0.36	−0.06	0.403^a^	**0.20**
Median (IQR)	1.96 (1.81-2.19)	2.01 (1.82-2.30)	(−0.20 to 0.08)		
Range	1.71-2.32	1.28-2.82			
**%ROS Positive**					
Mean ± SD	44.7 ± 8.4	74.9 ± 6.7	−30.2	**<0.001^a^**	**3.91**
Median (IQR)	42.5 (39.8-46.3)	76.0 (71.9-80.3)	(−33.5 to −26.9)		
Range	29.9-75.2	58.2-85.1			
**% Phagocytosis**					
Mean ± SD	45.0 ± 10.2	75.9 ± 5.5	−30.9	**<0.001^a^**	**3.65**
Median (IQR)	42.1 (39.9-45.6)	78.4 (73.5-80.3)	(−35.0 to −26.8)		
Range	35.2-82.1	63.4-84.9			

a: Independent t-test; p < 0.001 (highly significant); CI = Confidence Interval; IQR = Interquartile Range; WBC = White Blood cell Count; ANC = Absolute Neutrophil Count; ROS = Reactive Oxygen Species.

Subgroup analysis of MM patients showed no significant differences in WBC (p = 0.703), ANC (p = 0.617), percentage reactive oxygen species positive (p = 0.290), or percentage phagocytosis (p = 0.134) across disease stages. Similar analyses by disease status and infection history also showed no significant differences in outcome variables Table 4.

**Table 4:** Subgroup analysis of variables by disease stage in multiple myeloma patients.

Variable	Stage I (n = 7)	Stage II (n = 13)	Stage III (n = 10)	p-value
**WBC (×10^9^/L)**				
Mean ± SD	4.45 ± 1.30	4.09 ± 0.78	4.10 ± 1.02	0.703^a^
Median (IQR)	4.43 (3.67-5.12)	4.01 (3.59-4.55)	3.96 (3.54-4.32)	
**ANC (×10^9^/L)**				
Mean ± SD	2.04 ± 0.29	1.95 ± 0.17	1.96 ± 0.19	0.617^a^
Median (IQR)	1.99 (1.88-2.23)	1.95 (1.81-2.08)	1.95 (1.85-2.10)	
**%ROS Positive**				
Mean ± SD	48.5 ± 13.4	42.1 ± 5.9	44.9 ± 6.9	0.290^a^
Median (IQR)				
**%Phagocytosis**				
Mean ± SD	52.5 ± 14.8	42.4 ± 6.5	42.5 ± 6.4	0.134^a^

a: Independent t-test; WBC = White Blood cell Count; ANC = Absolute Neutrophil Count; ROS = Reactive Oxygen Species.

The WBC showed a negligible effect (mean difference = 0.14 × 10^9^/L, 95% CI: −0.31 to 0.59, Cohen’s d = 0.15), and the ANC demonstrated only a small effect (mean difference = −0.06 × 10^9^/L, 95% CI: −0.20 to 0.08, Cohen’s d = 0.20). In contrast, functional immune parameters, as presented in Table 5, revealed substantially different outcomes. The MM group exhibited very large and clinically significant reductions in both reactive oxygen species-positive cell percentages (mean difference = −30.2%, 95% CI: −33.5 to −26.9, Cohen’s d = 3.91) and phagocytosis activity (mean difference = −30.9%, 95% CI: −35.0 to −26.8, Cohen’s d = 3.65) compared to controls.

**Table 5:** Effect sizes and clinical significance of outcome variables.

Outcome Variable	Mean Difference	95% CI	Cohen’s d	Interpretation
(MM – Control) of Difference				
WBC (×10^9^/L)	+0.14	−0.31 to 0.59	0.15	Negligible
ANC (×10^9^/L)	−0.06	−0.20 to 0.08	0.20	Small
%ROS Positive	−30.2	−33.5 to −26.9	3.91	Very Large
%Phagocytosis	−30.9	−35.0 to −26.8	3.65	Very Large

Cohen’s d interpretation: 0.2 = small, 0.5 = medium, 0.8 = large, >1.2 = very large; WBC = White Blood cell Count; ANC = Absolute Neutrophil Count; ROS = Reactive Oxygen Species.

Fig 2 Shows individual test results for phagocytic and reactive oxygen species-positive neutrophils, plotted in GraphPad Prism Version 5. The mean percentage of phagocytic neutrophilsi was 45.0% in patients with MM and 75.9% in healthy controls (p < 0.0001). Likewise, the mean percentage of reactive oxygen species-positive neutrophils was 44.7% in patients with MM and 74.9% in healthy controls (p < 0.0001).

**Fig 2:**
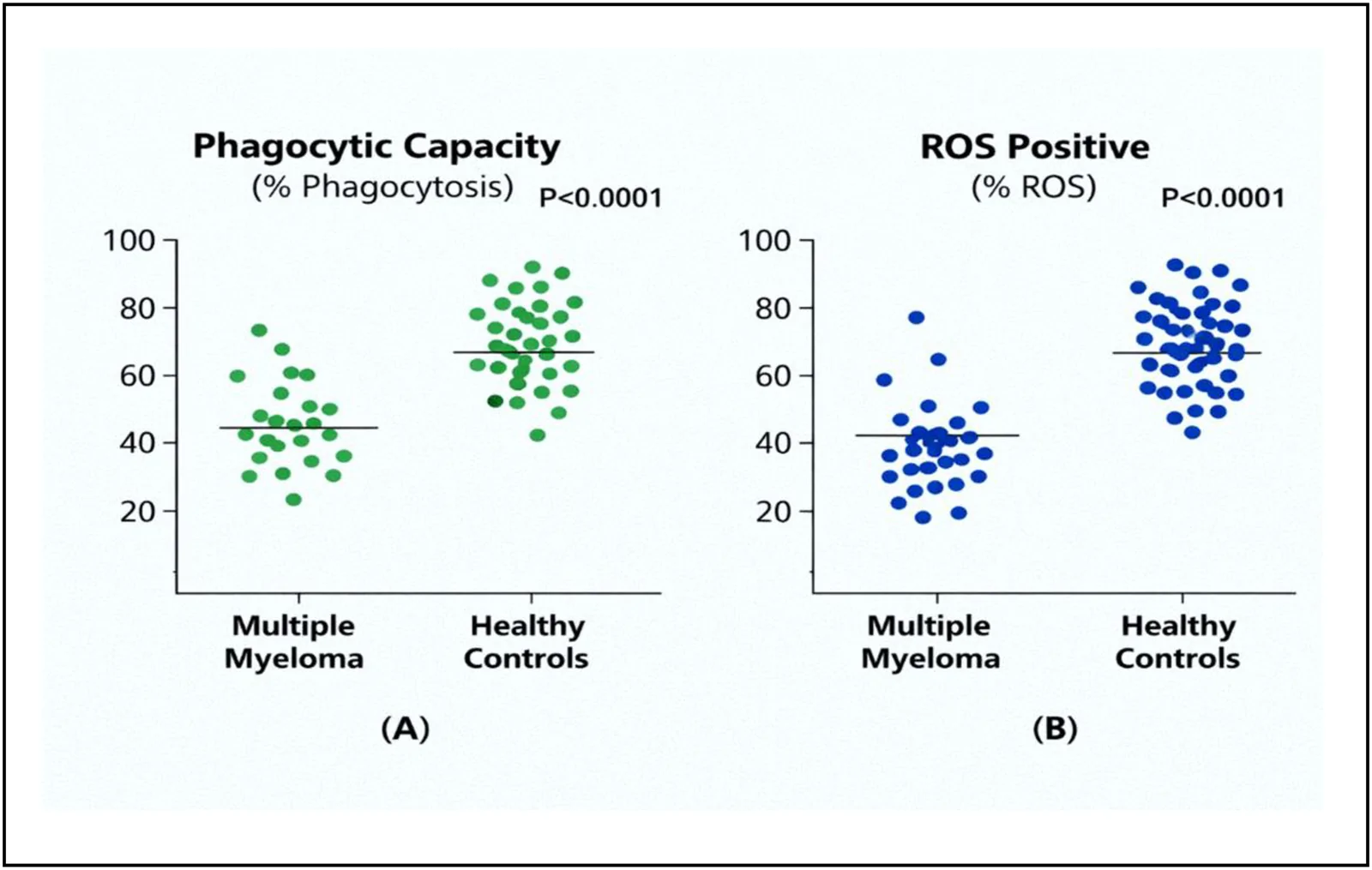
Comparison of neutrophil functional parameters between multiple myeloma patients and healthy controls. (A) Shows phagocytic capacity (% phagocytosis), while (B) shows the proportion of reactive oxygen species-positive neutrophils (% ROS). Each point represents an individual participant. The horizontal line indicates the mean value for each group. Significance is denoted by (p < 0.0001).

### Logistic regression analysis

Binary logistic regression analysis showed that MM was significantly associated with reactive oxygen species dysfunction (OR=5097.6, 95% CI: 158.9–163,542.1, P < 0.001). The association was even stronger for phagocytic dysfunction (OR=7272.7, 95% CI: 208.5–253,866.5, P < 0.001). In contrast, age, sex, WBC, and ANC were not significantly associated with either reactive oxygen species or phagocytic dysfunction (all P > 0.05), Tables 6 & Table 7 7.

**Table 6:** Logistic Regression Analysis – reactive oxygen species production dysfunction.

Variable	B	SE	Wald χ^2^	P-value	OR	95% CI
**Multiple myeloma**	**8.542**	**2.384**	**12.84**	**<0.001**	**5097.6**	**(48.2-539,180)**
Age	0.012	0.028	0.19	0.666	1.01	(0.96-1.07)
Sex (Male vs Female)	−0.634	0.856	0.55	0.459	0.53	(0.10-2.82)
WBC	−0.289	0.394	0.54	0.464	0.75	(0.35-1.62)
ANC	0.418	0.982	0.18	0.670	1.52	(0.22-10.48)
Constant	−7.824	3.126	6.26	0.012		

Model χ^2^ = 54.36, df = 5, P < 0.001; Nagelkerke R^2^ = 0.823; Overall Classification Accuracy = 95.8%; WBC = White Blood cell Count; ANC = Absolute Neutrophil Count.

**Table 7:** Logistic Regression Analysis – phagocytosis activity dysfunction.

Variable	B	SE	Wald χ^2^	P-value	OR	95% CI
**Multiple myeloma**	**8.891**	**2.512**	**12.52**	**<0.001**	**7272.7**	**(59.4-890,542)**
Age	0.018	0.029	0.37	0.541	1.02	(0.96-1.08)
Sex (Male vs Female)	−0.782	0.901	0.75	0.385	0.46	(0.08-2.67)
WBC	−0.245	0.401	0.37	0.541	0.78	(0.36-1.71)
ANC	0.124	1.045	0.01	0.906	1.13	(0.15-8.71)
Constant	−8.456	3.289	6.61	0.010		

Model χ^2^ = 56.12; WBC = White Blood cell Count; ANC = Absolute Neutrophil Count.

## Discussion

This study examined neutrophil functionality in patients with MM compared with healthy controls, focusing on phagocytic activity and reactive oxygen species production. Although neutrophil counts were largely maintained, significant functional impairments were present, including reduced phagocytosis and diminished reactive oxygen species production in patients with MM. These data suggest that qualitative alterations in neutrophil function may occur alongside preserved neutrophil counts in this cohort. Nevertheless, these results should be interpreted as evidence of an association rather than definitive proof that neutrophil dysfunction alone accounts for the increased susceptibility to infection observed in MM.

ANC were preserved, consistent with Romano [9]. This finding is significant despite impaired functional capacity, as standard hematological indices may not fully reflect innate immune competence. In this study, the ANC did not differ significantly between patients with MM (mean: 1.98 × 10^9^/L) and controls (mean: 2.04 × 10^9^/L; p = 0.403), whereas assays of phagocytic activity were reduced by an average of 30.9% (%phagocytosis 45.0 vs. 75.9; p < 0.001) and reactive oxygen species production was diminished by approximately 30.2% in the patient group (% reactive oxygen species 44.7 vs. 74.9; p < 0.001). These results show that functional assessment may yield additional insights beyond conventional quantitative measures in selected populations.

The mechanisms responsible for impaired neutrophil function in MM are likely complex and multifactorial. The bone marrow microenvironment in MM is defined by persistent inflammatory signaling, abnormal cytokine production, and interactions between malignant plasma cells and immune effector cells, all of which may influence neutrophil maturation and activity. Prior research has reported abnormalities in neutrophil phenotype, including modified expression of fragment crystallizable (Fc) gamma receptors, with increased expression of Fc gamma receptor I (FcγRI) and decreased expression of FcγRIIIa, which may impair pathogen clearance [9], and increased expression of programmed death-ligand 1 (PD-L1), a molecule known to suppress cell responses and promote immune evasion by tumors [20]. Elevated concentrations of NETs or their components have also been documented in neutrophils from patients with MM [22,23]. These findings align with the present results, which show reduced phagocytic activity and diminished reactive oxygen species production, providing functional evidence that complements these phenotypic observations. However, the exact molecular pathways underlying the dysfunction remain to be elucidated.

The considerable reduction in reactive oxygen species production observed in patients with MM, consistent with Askman’s findings [34], is noteworthy. Reactive oxygen species production is an essential component of the neutrophil oxidative burst and is required for killing intracellular pathogens. Impaired production may result directly from disease-related immune dysregulation, including alterations in neutrophil signaling pathways induced by the tumor microenvironment. Additionally, antimyeloma therapies, such as immunomodulatory drugs, proteasome inhibitors, corticosteroids, and prior chemotherapy, may affect neutrophil function [27]. However, because no participants were receiving active treatment at enrollment, treatment-related effects are unlikely to account for the observed reduction in reactive oxygen species production.

The reduction in phagocytic activity observed in the MM group may further impair innate immune responses. Although phagocytosis and reactive oxygen species production are distinct neutrophil functions, they are biologically interconnected processes that jointly enable pathogen recognition and elimination. In this study, logistic regression analysis demonstrated strong associations between MM and both reactive oxygen species and phagocytic dysfunction. By contrast, age, sex, WBC, and ANC were not significantly associated with these outcomes.

Another clinically relevant observation was the high proportion of patients reporting a recent history of infection. Although this study was not designed to evaluate infection outcomes or establish a causal relationship between neutrophil dysfunction and infection risk, the coexistence of frequent infections and impaired neutrophil functional parameters supports the need for further study. Infection susceptibility in MM is recognized as multifactorial, involving not only neutrophil abnormalities but also defects in humoral immunity, impaired cellular immunity, complement dysfunction, and the immunosuppressive effects of therapy [10,35]. Accordingly, the present findings should be considered as one aspect within the wider context of immune dysregulation associated with MM.

Subgroup analyses revealed no statistically significant differences in neutrophil function across disease stages, disease status categories, or recent infection history. The main objective of this study was to assess neutrophil functional parameters in patients with MM, rather than to determine disease progression or evaluate clinical outcomes. Accordingly, future research involving larger, prospectively followed cohorts will be necessary to clarify the relationship between neutrophil dysfunction, disease evolution, treatment exposure, and infection-related morbidity.

### Limitations

Several restrictions must be considered when interpreting the results. First, the cross-sectional design precludes establishing causal relationships between MM and neutrophil dysfunction. As a result, it remains unclear whether the observed impairments in phagocytic activity and reactive oxygen species production are direct consequences of the disease process, previous treatment exposure, or other factors.

Second, although post hoc power analysis suggested that the achieved sample size provided adequate power for the primary outcomes, the planned sample size was not attained. Recruitment was constrained by strict eligibility criteria intended to minimize confounding factors that could affect neutrophil function. Among the MM patients, two otherwise eligible participants were excluded because of a chronic inflammatory condition and an uncontrolled metabolic disorder. Similarly, among healthy controls, eight individuals were excluded due to corticosteroid use and three due to uncontrolled metabolic disorders. While these exclusions reduced the number of participants available for analysis, they strengthened the study’s internal validity by limiting the influence of conditions and treatments known to affect neutrophil activity. Nevertheless, the relatively small sample size, particularly in subgroup analyses, may have limited the ability to detect subtle differences by disease stage, disease status, or recent infection history. Consequently, non-significant subgroup findings should be interpreted with caution.

Finally, the single-center design may limit the generalizability of these outcomes to broader MM populations. Larger multicenter longitudinal studies are required to confirm these results and to further investigate the relationships among neutrophil dysfunction, treatment exposure, disease progression, and infection-related outcomes.

## Conclusions

This study shows that patients with MM have significant neutrophil dysfunction, defined by diminished phagocytic activity and diminished reactive oxygen species production, despite ANC comparable to those of healthy controls. These results suggest that conventional quantitative hematological measures alone may not adequately reflect innate immune competence in MM, and that qualitative functional defects may contribute to the wider spectrum of immune dysregulation associated with the disease.

The observed associations between MM and neutrophil dysfunction support the growing body of evidence that alterations in innate immunity may play an important role in the elevated vulnerability to infection in these patients. However, given the cross-sectional design and relatively small sample size, interpret the findings as indicative of an association rather than evidence of a causal relationship. In addition, susceptibility to infection in MM is multifactorial and goes beyond neutrophil abnormalities alone.

This study spotlights the potential value of incorporating functional neutrophil assessment into immunological investigations of MM. Larger longitudinal studies are needed to examine the mechanisms underlying neutrophil dysfunction further and to determine its relationship with disease progression, treatment exposure, and infection-related clinical outcomes.

## Supporting information

S1 Fig Representative flow cytometric gating strategy used for identification prior to functional analyses. Neutrophils were identified and gated based on their characteristic forward scatter and side scatter properties, distinguishing them from other white blood cells.
(PDF)

S2 Fig Flow cytometry analysis of neutrophil phagocytosis using pHrodo^TM^ BioParticles^®^. Flow cytometry analysis showing red-stained neutrophils that phagocytosed pHrodo^TM^ BioParticle^®^ and grey unstained controls. The right-shifted red fluorescence peak confirms particle uptake and acidification after incubation at 37 °C compared with the ice control.
(PDF)

S3 Fig Flow cytometry analysis of neutrophil reactive oxygen species production using DCFH-DA. Neutrophils were gated by forward and side scatter and analysed for DCF fluorescence in the FITC channel (488 nm/525 nm). Overlaid histograms show increased fluorescence in *E. coli-*activated neutrophils compared with controls, confirming reactive oxygen species production.
(PDF)

S4 File De-identified summary of demographic and assay results. This file contains de-identified participant-level demographic and neutrophil functional assay data that underlie the results reported in the manuscript. Direct participant identifiers have been removed. Age is reported in whole years, and categorical variables are coded as specified in the accompanying data dictionary.
(XLSX)
