# Supplementary material for: Functional characterization of neutrophils in patients with multiple myeloma
Source: PLoS One. 2026 Oct 8;21(10):e0360063. doi: 10.1371/journal.pone.0360063 (PMC13649033; doi:10.1371/journal.pone.0360063)
Supplement: Supplementary file 1 [file pone.0360063.s001.pdf]

S1 Fig. Representative flow cytometric gating strategy used for identification before functional analyses.

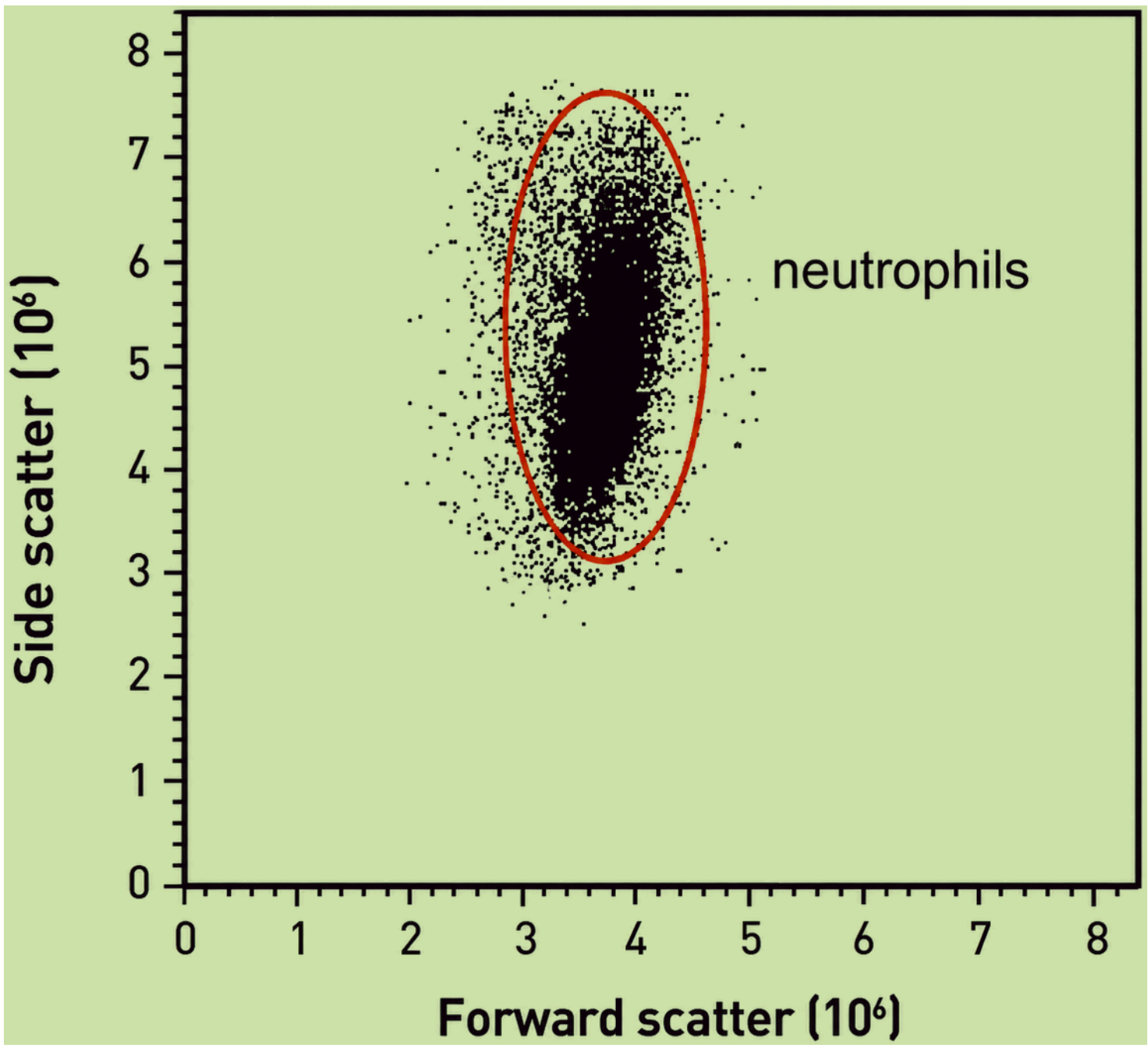

S1 Fig. Shows neutrophils identified based on their characteristic forward scatter (FSC) and side scatter (SSC) properties. The neutrophil population was manually gated (red ellipse) on the FSC versus SSC dot plot according to its distinct size and granularity. This gated population was subsequently used for downstream functional assays, including phagocytosis and reactive oxygen species production analyses.
