# Supplementary material for: Functional characterization of neutrophils in patients with multiple myeloma
Source: PLoS One. 2026 Oct 8;21(10):e0360063. doi: 10.1371/journal.pone.0360063 (PMC13649033; doi:10.1371/journal.pone.0360063)
Supplement: Supplementary file 2 [file pone.0360063.s002.pdf]

S2 Fig. Flow cytometry analysis of neutrophil phagocytosis using pHrodo™ BioParticles®.

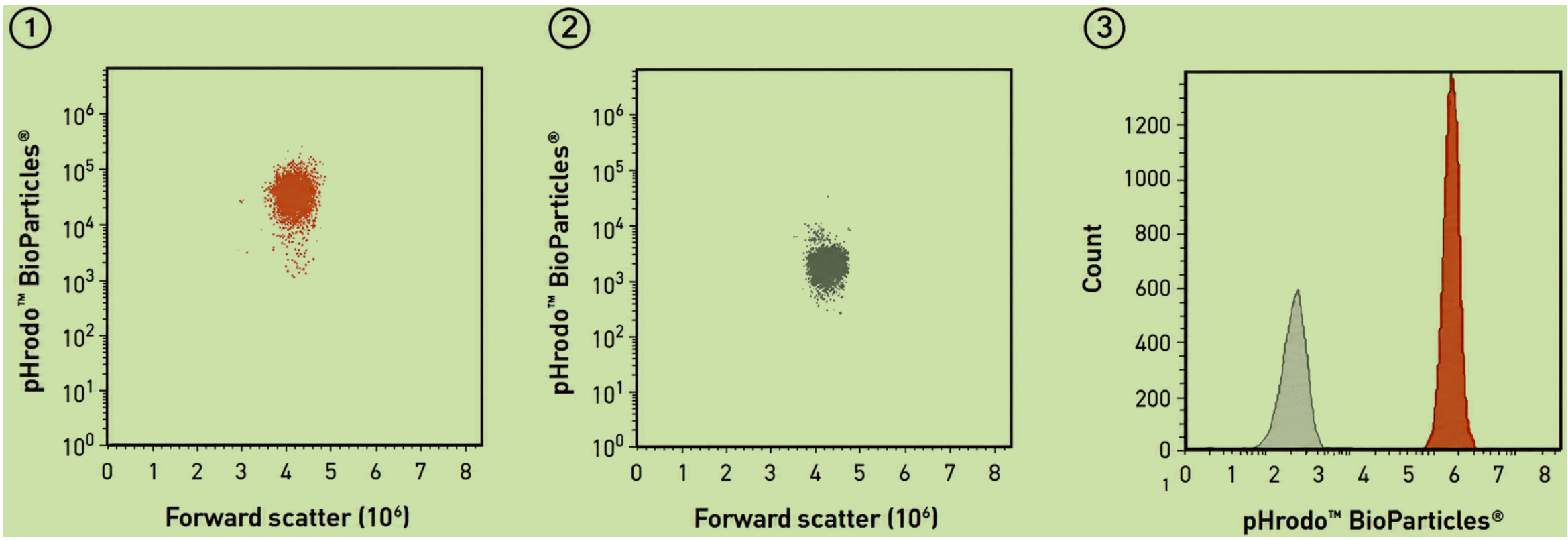

S2 Fig. (1) shows neutrophils that have phagocytosed pHrodo™ BioParticles™ conjugate, (2) A control sample in which phagocytosis was inhibited by incubating on ice, demonstrating the lack of fluorescence because pHrodo™ BioParticles™ conjugates were not in acidic phagosome compartments, and (3) A histogram overlay of (1) and (2) showing the fluorescence separation between the experimental and negative control sample.
