# Supplementary material for: Functional characterization of neutrophils in patients with multiple myeloma
Source: PLoS One. 2026 Oct 8;21(10):e0360063. doi: 10.1371/journal.pone.0360063 (PMC13649033; doi:10.1371/journal.pone.0360063)
Supplement: Supplementary file 3 [file pone.0360063.s003.pdf]

S3 Fig. Flow cytometry analysis of neutrophil reactive oxygen species production using DCFH-DA.

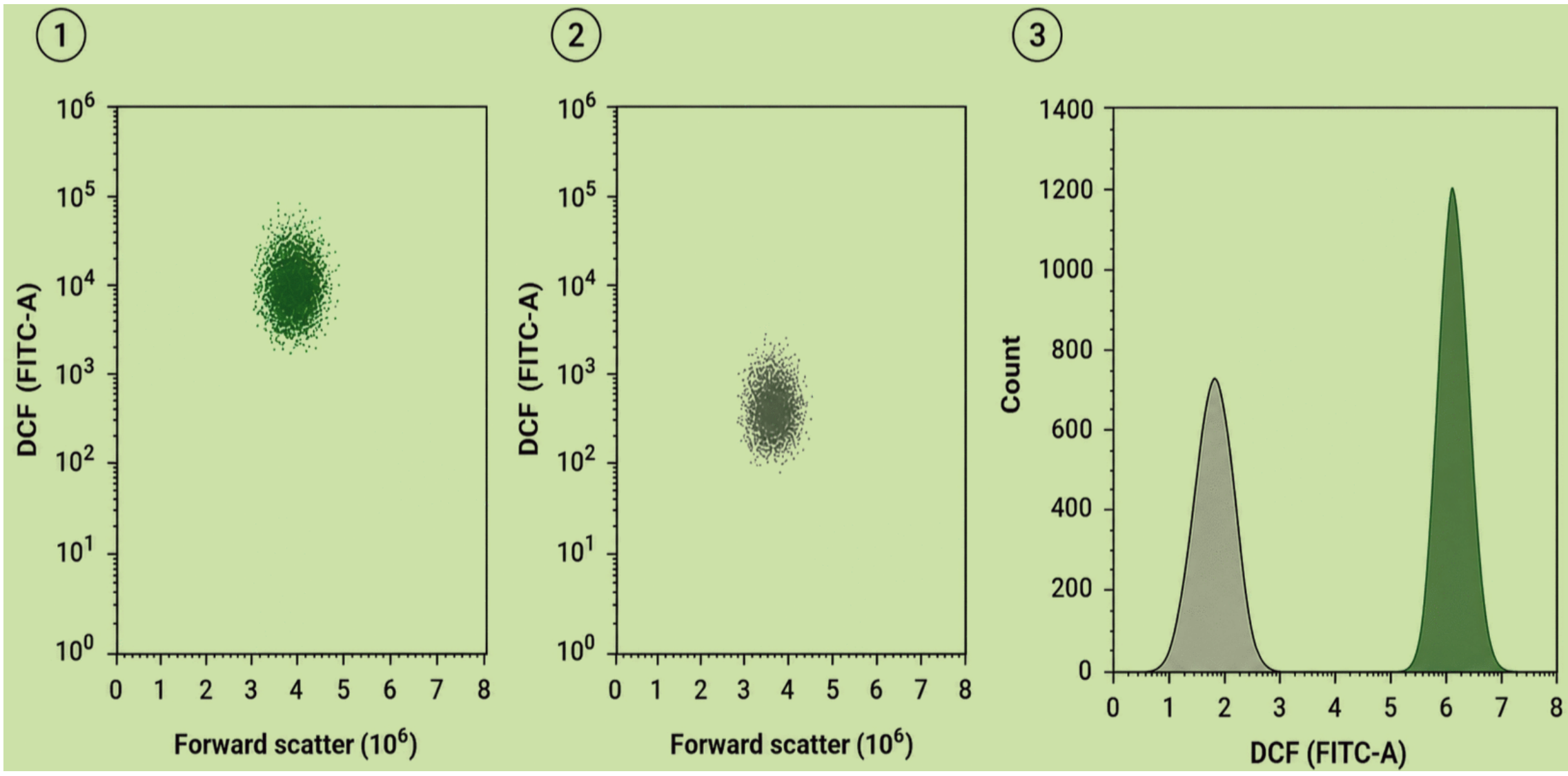

S3 Fig. (1) shows a positive control; neutrophils show strong green fluorescence in the FITC channel, indicating high reactive oxygen species production (DCF^+ 95.6%). (2) Show a negative control, neutrophil show minimal fluorescence (DCF^+ 1.2%). (3) Show a histogram: the green peak (positive control) shifts rightward relative to the grey peak (negative control), confirming increased DCF fluorescence after stimulation. Neutrophils were activated with heat-killed *E. coli,* incubated with 10 μM of DCFH-DA, and reactive oxygen species production was measured as DCF fluorescence on a CytoFlex flow cytometer.
